# *Urip iku urup* (life is lit) by service to others: a qualitative study of frontline healthcare workers’ lived experiences providing patient care in Indonesia’s COVID-19-designated hospital

**DOI:** 10.1186/s12913-023-09257-2

**Published:** 2023-03-16

**Authors:** Ayu Puspita Ningrum, Malene Missel

**Affiliations:** 1 Department of Public Administration, Faculty of Social and Political Sciences, Universitas Airlangga, Surabaya, Indonesia; 2 Department of Cardiothoracic Surgery, Rigshospitalet, Copenhagen University Hospital, Copenhagen, Denmark

**Keywords:** Lived experiences, Frontline healthcare workers, Patient care, COVID-19, Coronavirus, Indonesia, Qualitative study, Hermeneutic phenomenology

## Abstract

**Background:**

While COVID-19 affects every walk of human life, it especially implicates healthcare workers at the forefront of the pandemic due to their vulnerable involvement in providing first-line treatment. This study presents the lived experiences of frontline healthcare workers serving in Indonesia’s COVID-19-designated hospital, one of the severely afflicted healthcare settings wherein resource challenges, public health crisis, and political constraints intersect as policy conundrums.

**Methods:**

Using a qualitative exploratory-descriptive approach, this study drew on thirteen in-depth, semi-structured interviews with frontline healthcare workers who have experiences providing first-line COVID-19 patient care in the COVID-19 hospital. The data analysis commenced with the verbatim transcription of the interview data, which was then subjected to a systematic thematic analysis employing hermeneutic phenomenological principles.

**Results:**

The exploration of the participants’ accounts reveals eight interconnected themes: facing resource scarcity and resignation; experiencing service-induced burnout due to occupational workload; encountering fears of being infected and infecting others; engaging in positivity through social connectedness; having dilemmas over healthcare rationing; developing negative emotions during patient interactions; coping through spirituality and religiosity; and embodying a life of service.

**Conclusion:**

Managing healthcare in resource-limited, crisis settings presents multifaceted challenges that exceed mere structural modifications, requiring prioritized public health investment to ensure optimal patient care. Therefore, healthcare policy development and implementation should equally emphasize the well-being of frontline healthcare workers to foster sustainable healthcare delivery and achieve improved patient outcomes.

## Introduction

While the COVID-19 pandemic appears to be “under control” in some Western countries, with many transitioning from an acute pandemic response to a sustained management strategy, there remains a significant prevalence of infections and deaths in others, such as in Indonesia. As one of the first countries to be affected by the first wave of the pandemic and on the current list of countries where COVID-19 infections stand significantly, Indonesia has been dealing with a continued overwhelmed and overburdened healthcare system due to its substandard worker-to-patient ratio [1].

Despite the recent development and distribution of vaccines [2], Indonesia still has the highest incidence of COVID-19 in Southeast Asia, with at least 17,261 active cases recorded as of 15 October 2022 [3], putting pressure on frontline healthcare workers to provide care for the affected [4]. This study contributes to improving the understanding of experiences learned from healthcare workers during the COVID-19 public health crisis, as well as to the translation of research findings into evidence-based policy and practice aimed at enhancing frontline healthcare workers’ well-being and welfare, and by extension, the global public health architecture.

## Background

COVID-19 has magnified the world’s economic geography and exacerbated existing social disparities, bringing attention to the fragile nature of global health governance. With the pandemic and its unprecedented effects, frontline healthcare workers have emerged as notable figures by delivering the very best healthcare services possible to alleviate the strains imposed on patient care [5, 6]. The pressures placed on the healthcare workforce to protect public health amid the pandemic, however, are nowhere more evident than in Indonesia. As of the confirmation of the first two cases, Jakarta continues to experience an enduring increase in confirmed COVID-19 cases. With the healthcare system in the city struggling to keep up with the rising rates of infection, the vulnerability for many COVID-19-infected population groups has created an unmanageable surge in hospital bed occupancy rates.

Amidst the intentions of averting the risk of infecting non-COVID-19 patients treated in the regular hospitals, the Indonesian government reconstructed a Jakarta apartment building in early March 2020 to accommodate the provisions of first-line hospitalizations for COVID-19 patients. Given that this emergency facility still functions as a central emergency hub for treating and caring COVID-19 patients, as well as the current COVID-19 figures in Indonesia, it is imperative to deduce implications for professionals in the healthcare sector and to share experiences learned from working in these challenging environments. In so doing, this study seeks to draw on the lived experiences and perspectives of frontline healthcare workers serving in Indonesia’s COVID-19-designated hospital.

## Methods

### Design and setting

This study is underpinned by an interpretivist paradigm [7, 8], whereby the insights are mainly derived from a phenomenological exploration that is not merely in the act of understanding particularities but in the system of meanings from which it is composed to the larger contextualities of the study. A qualitative exploratory-descriptive approach [9] further inspired the research process with a focus on exploring and describing the experiences as they were lived by frontline healthcare workers. In employing this design, the study was informed by the work of van Manen’s hermeneutic phenomenology [10–15]. The approach presupposes an attentive attunement and open-mindedness that offers rich textual information about the “human” side of a phenomenon and enables interpretation of symbolic order meanings.

With a three-month research period commencing in June 2021 and concluding in August 2021, this study took place in one of (and the largest of) Indonesia’s first-line COVID-19-designated hospitals in Jakarta. As a leading hospitalization for COVID-19 patients in Indonesia, this makeshift hospital was reconstructed in early March 2020 and is headed by a number of significant government and non-state entities, including (among others) the Indonesian military, police, ministry, and medical association. Built on a 10-hectare site with roughly 7,000 apartments in ten buildings, the hospital is one of the world’s largest COVID-19-focused hospitals with a total housing capacity of approximately 22,000.

During the pandemic in Indonesia (March 2020–as of this writing), four out of ten apartment buildings were converted into COVID-19-designated facilities, with zoning implemented to prevent cross-infection and the hospital area divided into three distinct localities: the red zone for COVID-19 patients, the yellow zone for the occupation and housing of healthcare workers, and the green zone for non-service-providing physical activities (such as sports, exercises, and recreational activities). Due to the emergency nature of the hospital and its very unusual working conditions, it is important to note that personnel service contracts are renewed on an as-needed basis, with them having the option to renew their contracts or being transferred to another COVID-19 emergency facility if so directed. At the time of the study, approximately 3,000 healthcare workers were employed in at least 25 departments located in both the yellow zone (administrative and secretarial services) and the red zone (direct patient care), and some personnel were routinely rotated monthly to other COVID-19-designated hospitals in other regions of Jakarta as part of contract renewals or military and police assignments, and others had their service contracts officially ended.

### Participants

Considering that purposive sampling is meaningful within the phenomenological notion of intentionality, this study invited frontline healthcare workers (*n* = 13) with various levels of expertise and responsibilities who have first-hand knowledge and prior experiences of providing direct patient care in the COVID-19-focused hospital in Indonesia. A range of snowballing was also employed to identify and recruit new participants among the existing study participants’ acquaintances, thereby proceeding in locating information-rich participants through this chain-referral approach.

Eight males and five females were included in this study, with the participants aged between 21 and 30 years (*n* = 7), 31–40 years (*n* = 3), and 41–50 years (*n* = 3). Of the participants, there were five medical personnel (three general practitioners and two pulmonologists), two clinical psychologists, four nurses, and two dieticians. Before their services in the COVID-19-designated hospital, most participants had worked for a range of 1–10 years (*n* = 10), 11–20 years (*n* = 2), and over 20 years (*n* = 1), but a sizeable majority has been serving in the hospital for over 12 months (*n* = 7), with the remainder serving for, respectively, 1–6 months (*n* = 3) and 7–12 months (*n* = 3).

### Data collection and ethical consideration

Following ethical approval from Universitas Airlangga research ethics board (Approval No. 2281/UN3.1.7/PT/2020), and with the purpose of getting rich and in-depth experiences, a semi-structured interview protocol consisting of open-ended questions, followed by probing questions, was developed to explore the participants’ lived experiences and perspectives of serving at the COVID-19-designated hospital, as well as their thoughts, concerns, and feelings.

After contacted participants clearly and freely expressed their consent to participate, the interviews were arranged with flexibility and agreement to both parties, also appropriately held in a location convenient to the participants. Before commencing the interviews, the participants were provided with an explanation of the research objectives, as well as hard copies of letters of information and informed consent to document the essential statement of agreement between two parties, with the former meant to be kept by the participants and the latter was meant for both sides.

van Manen’s phenomenological approach of being open and giving time and space for the participants to talk about their experiences was valuable in exploring insights [15]. The in-depth, semi-structured interviews started in exploratory modes and progressed toward a more in-depth manner, with questions exploring participants’ descriptions of their sense of caring for and treating patients during the COVID-19 trajectories, recollections of patient and family encounters, experiences of working in the emergency facility hub, as well as key driving causes for service.

The interviews were audio-recorded, lasted for approximately 45–90 min, and conducted in a face-to-face format so long as adequate health protocols were adhered. Equally important, observational notes were engaged throughout the interviews, with memoing and reflexive journaling were also used afterward to support observations of participants’ behaviors, reactions, and emotions during the interviews. This journaling particularly provided an attunement of the interview moments; thus, letting the phenomena of the lived experiences and perspectives of frontline healthcare workers be disclosed.

### Data analysis

In a dynamic process, van Manen’s analytical steps, holistic, selective, and detailed readings, led the way in exploring the participants’ experiences [14]. The data analysis process started with a review of the interview transcripts purposefully attending to embedded meanings [12], with both authors reflecting and discussing data and interpretations at all levels in the analytical process in order to ensure trustworthiness of the findings. An iterative, inductive approach of analysis was used to study the transcripts which were clustered and analyzed in order to identify understanding and meaning of the material as a whole. In a process of dwelling in and distancing, which also included continuously going back and forth between clusters of meaning and the interview text as a whole, clusters were grouped into essential themes to capture the phenomenon of interest.

Indeed, as highlighted by van Manen [11], the process of writing and rewriting is essential because it helps researchers to transcend beyond the identification and comparison of themes to a fuller understanding of the meaning of pre-reflective experiences both within and across transcripts [15]. Following this step, thematic reflections were described as figures of meaning to help point to possible eidetic meaning aspects of the frontline healthcare workers’ experiences, containing detailed descriptions of lived experiences with an aim of illuminating the complexity and structure of meaning and will serve as a structure in the presentation of findings.

## Results

Hearing the participants’ voices in this study enabled it to investigate as well as to explore the connections, patterns, and recurring themes that reflect the experiences of the participants as a whole. The findings of eight interconnected, essential themes were articulated by participants’ frank, pre-reflective and honest expressions and reactions in the profound sense of emotionally-attuned experiences; described in detail below. This section also presents empirical data, i.e., interview quotes from participating healthcare workers, not to provide proper proof or argumentation but as examples that amplify the nearness and presence of the frontline healthcare workers’ understanding of their experiences.

### Succumbing to the system asymmetry

The participants expressed their confusion and dissatisfaction with their early interactions with the COVID-19-designated hospital, highlighting the rather non-existent system and absence of infrastructure that serves as the foundation of an emergency facility. Due to the absence of a well-established system and the inadequacy of facilities, a great level of ingenuity had to be exercised to adapt to its ever-changing emergency nature. Further, participants were concerned about the inadequacy of quality human resources in each of their departments, which affected them emotionally and induced *jenuh*-like emotions (getting weary of the situation). These emotions, manifested mostly as emotional exhaustion and overwhelm, were particularly present due to the strain to quickly adapt to a succession of personnel changes and their distinct work styles in a short time span, a situation likely linked to the hospital’s frequent personnel changes and constant contract renewals.

However, in addition to the feeling of *jenuh*, participants in leadership positions regarded the system as problematic when they had to assist and instruct new personnel, as they perceived it as impeding their previous progress. Dissatisfaction and frustration with the system was also expressed, as it necessitated further adaptations to begin anew and ingenuity to manage the system. For example, because the psychology department is led by a military member and there is a rotational command arrangement, the monthly leadership changes made senior clinical psychologists especially sensitive and frustrated when the new leadership was incompatible with their membership.

Despite these effects, participants became receptive to and attuned to the system’s inadequacies, succumbing to them either out of habitual familiarity or a sense of powerlessness. Even for nurses in the COVID-19 swabbing department, who were particularly exposed to the frightening state of encountering patients and faced greater risks in their patient encounters, this system asymmetry pertaining to institutional facilities was not only germane; it also compelled nurses to (powerlessly) concede defeat to the hospital’s flawed system.

*At one time, the room for collecting COVID-19 swabs was inadequate, as there were no windows and thus no air circulation. I was really frustrated when my teammates and I sought a different room but were never provided with one, possibly due to its inaccessibility. Finally, I can only adapt; I can only ngarepin [depending on] the air conditioner and fan in the room.*

### Becoming subjected to burnout

As the principal treatment facility for COVID-19 patients in Indonesia, the workload experienced by those at the forefront of the pandemic is indeed unfathomable. Although most departments require workers to work in the red zone and visit patients for only eight hours before a 32-hour break, the workload during the eight-hour visit is rather demanding and even stressful.

Two participants discussed the challenging workload allotted to them as clinical psychologists, which includes counselling patients, interviewing prospective personnel, and organizing events for patients (such as webinars and fun games). During therapy sessions in particular, they often felt as if they were absorbing the patients’ negative energy owing to the immense stress of listening to many (patients’) anxieties and complaints. Despite this, both participants maintained the same caring and supportive approach to the patients: *“We are here to serve patients, and they need us”*, and attempted to recall the joy they experienced when patients’ conditions improved.

However, due to a different system in the swabbing department that necessitates healthcare workers to work every day from 8 a.m. to 5 p.m., two nurses described the stressful circumstances of swabbing 600–700 people in a single day. Both nurses also mentioned their exhaustion as a result of the hospital’s mass swabbing activity, which they described as: *“…from morning to evening, we must swab every healthcare worker [about 3,000 people]”*. They also expressed burnout, especially when swabbing challenging patients, such as those with ADHD or bodies of those who died in the ICU, leaving them emotionally and physically exhausted due to their department’s lack of human resources, but with a strong sense of duty to perform their job.

### Having fears of being infected and infecting

COVID-19 was a significant source of concern for the participants, prompting them to devise a variety of precautions to reduce their risk of infection. Concerns about the new COVID-19 strain particularly prompted participants, in their own ways, to maintain their level of safety when providing care to COVID-19 patients.

In an intriguing twist, participants stated that their fears of infection stemmed from a desire to prevent infecting others; for some, this fear manifested as an unwillingness to return home. In fact, the COVID-19 hospital’s system discourages and even prohibits workers from returning home, and all participants agreed that this is a prudent policy that must be followed despite their overwhelming desire to do so. Even during the two most important religious holidays celebrated by Muslim participants, they chose not to return home for fear of contracting their families and relatives and felt bitter toward those who could return in contrast to their peculiar situations.

Notwithstanding so, even participants who were able to return home (for urgent reasons) were not necessarily welcomed by their neighborhoods, indicating that their community was not as receptive to their presence after their services in the COVID-19 hospital. In this, participants expressed feelings of exclusion from their neighbors just because they worked in the COVID-19 hospital, with them further voicing their resentment at having to morally provide care for patients who ironically stigmatized them.

*My mother once prohibited me from returning home because she did not want me to become bahan omongan [the talk of the town]. I felt compelled to beg her, “Ya Allah, Ma! I will bring my COVID-19 test as proof!” I was also disappointed when my neighbors believed that working here is so terrifying... like, ‘she works in a COVID-19 hospital, she must be a carrier of the virus’, while in reality, who helps them when they are in need or do not follow to health protocols? It is us [healthcare workers].*

### Growing a sense of relatedness with patients

Participants, especially those who had been diagnosed with COVID-19 in the course of their service, exhibited a sense of connectedness and relatedness to their patients. Some even extended this by comparing patients in a COVID-19 hospital to those at conventional hospitals by stating that patients are essentially alone (for self-isolation purposes), and as COVID-19 hospital residents, their shared experience of being *“alone and far from my family”* enhanced their ability to relate with their patients.

One participant mentioned that she frequently felt irritated when she was tired or when she encountered demanding patients, but she always consoled herself with thoughts such as: *“I must consider my patients’ feelings because I am sure they do not want to be like this either”*. Another participant, who was particularly drawn to young children whom she described as *“reminding me of my lovely children back home”*, described swabbing a 14-day-old infant after the grandfather tested positive for COVID-19 as the most traumatic experience she could endure because *“the baby was crying so intensely because the Dacron swab was the same as those used for adults”*.

Participants with leadership and management responsibilities went even further by emulating a sense of connection with patients and instilling in their colleagues the importance of *“caring for patients as if they were our family”*, stating that *“it is impossible for us to hate our COVID-19 patients”*, despite experiencing emotional distress as a result of treating them. Participants understood that many patients may have had fears and anxieties following their initial COVID-19 diagnosis, and they felt motivated to ease their burdens. One participant continued by stating that he enjoyed conversing with his patients and joking with them to lighten their moods, especially with patients his age and the elderly because they tend to have the most negative thoughts about health deterioration and even death, and he felt obligated to share his empathy with them and foster a sense of togetherness.

### Facing dilemmas with healthcare rationing

Participants expressed concerns with the arbitrariness of discretion over (especially) healthcare rationing, comparing it to *“playing God without God’s power”*. One general practitioner detailed how, in a single day (8-hour shift), she and her teams had to cope with five consecutive deaths, followed by the next day of dealing with the same situation and receiving reports from nurses that the consciousness of several patients had weakened. She felt as though *“it was a marathon of patient deaths”* and felt bad for “maybe” lacking the knowledge necessary to save her patients. Yet, as she continued to speak in a conflicting manner, *“We lacked ventilators”* and *“All we can do is have a strong attitude and effective coping methods to cope with it”*. She further sought to portray her experiences in encountering dilemmatic healthcare rationing, recounting an agonizing and awful moment during COVID-19’s second wave peak in Indonesia when she was compelled to pick which patients should be taken off ventilators.

*We have a significant number of ‘ugly’ [deteriorating] patients and must choose which one should be prioritized. For example, we must determine whether a young patient and an elderly patient have comorbid conditions. Because there are so many elderly patients in the ER, if the elderly patient suffers kidney failure, hypertension, or diabetes, and the young patient is generally healthy, we must prioritize the young patient. At some point, we had to care for at least three patients at the same time, assessing their odds of survival, attempting to save them, or simply waiting and watching.*

Her experiences and emotions were also shared by participants with decision-making responsibilities, such as pulmonologists, recalling a moment when they, as physicians with discretion and autonomy, had to determine which patient should be transferred to the ICU. They proceeded by stating how they felt conflicted when they were required to choose which patients to provide prescriptions to due to a shortage of availability, and how they felt remorseful that they had to “prioritize” patients with a higher chance of survival and “put aside” others with less. In the end, they expressed remorse that, despite their extensive experience and knowledge, they had yet to contribute to what they perceived to be their expectations, and particularly developed a sense of guilt when they had to eventually face the patients’ families, explaining that *“breaking the news is a skill acquired the hard way”*. A general practitioner with a leadership position in his department, pondered on the consequences of his colleagues’ delivery of bad news when he tried connecting it to his experience.

*At times, we feel sad when the families of our patients condemn us, asking, ‘Why are you not saving my family?’ At one point, a patient’s family approached me begging for help; but, due to the hospital’s overcrowding, I suggested the family seek care elsewhere with better facilities and resources. A few days later, the family sent me a message stating, ‘Thank you for your advice. My family has died’, as if implying and persuading me that it was my fault and my cause, when I thought I had done all possible.*

### Developing negative emotions in patient interactions

Caregiving for sick persons entails vulnerabilities, as seen by the participants’ sentiment of not knowing what will happen next and, if they do, being unable to help. A participant who worked as a dietician in the ICU described how she was frequently confronted with the *“unimaginable and unexpected”* as patients’ conditions deteriorated and frequently culminated in death. She stated her sorrow and disappointment that, *“A few days ago, the patient was still able to drink the milk I gave her”*, as well as her anxieties and feelings of guilt that, *“Maybe it was my fault; perhaps I gave the patient too little milk, or I should have increased the amount… I am afraid that the patient’s condition is a result of my administering an incorrect dose”*.

As it turns out, such an experience is a collected encounter shared by the participants, including two clinical psychologists who felt burdened by the notion that they were powerless to assist a patient who attempted suicide in the building. In consequence, one psychologist even had reached the point where she sought consolation and emotional support from her colleagues, further expressing her feelings of frustration, regret, and powerlessness when she was confronted by her patient’s emotionally charged verbal remarks:

*On my first visit to the red zone, I encountered a patient whose mental instability demanded transfer to another facility. The patient, whose condition was already improving and stabilizing as a result of my treatment sessions, felt misled and offended that she had to be transferred to a different institution after having been accustomed to this one. I still remember her saying, ‘Perhaps I would still be here if I had not consulted with you. I should not have consulted with you’.*

But as the participants had also pointed out, they were not always in control of the patients’ care and (most of the time) had to follow on what was directed as the procedure, that there are higher levels of decision-making over which they have no control. This lack of professional autonomy affected a general practitioner, who, despite maintaining a fairly nonchalant demeanor throughout the interview, also had an emotional moment when he recalled an episode of feeling inadequate and incapable, “*It turns out my scope is just this much”*.

### Coping through spirituality and religiosity

In light of the significant adversities faced by participants, they recognized the importance of protecting their health and well-being through positive health behaviors and micro-strategies. While all participants acknowledged the significance of pragmatic considerations during their service at the hospital, some particularly highlighted the importance of spirituality and religion as a springboard for mending and health management. One participant narrated an excellent example of both aspects:

*Good people are those who can benefit others, my faith teaches. It is a tenet of my faith that I take to heart, and it is what drives me to help other people and what makes me happy while I am helping them. While it is true that we can only do so much on our own, I still think God is on our side whenever we do right. Obviously, we need to keep up our standard of healthful living. But I believe that if we act with our hearts, God will reward us with our hearts as well.*

Participants concurred with the preceding narrative and emphasized the importance of conforming to the teachings of their religion when they feared contracting the virus. Likewise, participants believed that their humanitarian calling would protect them, *“Insya Allah, if we maintain a high level of safety, our health will be protected, and we will not contract the virus. However, even if we contract COVID-19, we must ikhtiar [an Arabic phrase for putting effort] on how to recover in order to resume our work”*.

Among the participants, one participant who is a pulmonologist with almost two decades of experiences, was possibly the most spiritually inspired by his faith. For him, since COVID-19 is indiscriminate in its infectivity, he hoped that if he understood this from a more nuanced spiritual perspective, or as he put it, *“It was fated that way”*, he would feel more at ease during the healing process. Even as a survivor of COVID-19 who contracted the virus twice and lost both of his parents to the virus, he could find solace in the support and religiosity of his religion, underlining the necessity of accepting that everything is predestined as fate.

Indeed, with a flurry of unfortunate events unfolding at an unprecedented speed and despite their best efforts, participants were compelled to surrender to the painful reality. Nevertheless, an interesting facet emerged from their accounts, as they found comfort in the presence of a higher power, characterized as *“the act of not knowing everything but knowing that something greater exists”*. Additionally, participants believed that striving to provide the best possible care to their patients while giving their utmost effort, and surrendering to a higher power, assisted them in accepting the limitations of reality and embracing the concept of *“ikhlas”* – the act of letting go – throughout the process. Ultimately, this brought them a sense of comfort and release from the burden of their inability to save every patient, enabling them to focus on the ones they could help.

### Finding meanings in life of service

Given the excessive workload and high prevalence of burnout, it begs the question of “why” frontline healthcare workers continue to devote their lives to serving patients in the COVID-19 hospital. As it unfolds, all participants have a sense of usefulness and a desire to contribute to society in order to find purpose in their lives. For instance, a military dietician remarked that his military assignment to the emergency facility is the first time in his 20 years of service that it coincides with his passion, expressing delight that his profession can be channeled through such service.

*As a soldier, I am honored and proud to have been entrusted with serving in this place in service of my nation. I am proud of myself since I believe that not everyone wants to come here and because I am prepared to take risks while others are not.*

One clinical psychologist, who was highly worried and even had psychosomatic episodes after her first interaction with the hospital, shared a similar emotion and even became philosophical in her motivation, using the Javanese proverb *“urip iku urup”* (life is lit), stating that she *“personally feels satisfaction and a warm sensation in my heart when I am able to help others”*.

Two participants who identified themselves as altruistic, emphasized the need of having a sense of purpose while helping others in order to do so with sincerity and selflessness, stating that they felt “called” to serve others. One participant even put considerable attention in citing this “calling”, stating that her profession as a nurse compelled her to serve in the COVID-19 hospital, despite contracting the virus twice during her time there.

## Discussion

Drawing on hermeneutic phenomenological analysis, this study is offering a novel insight into frontline healthcare workers’ lived experiences and perspectives when providing patient care in Indonesia’s COVID-19-designated hospital. Further contributing to the burgeoning literature on the phenomenological studies of COVID-19, the eight overarching themes revealed by this study’s documentation of healthcare workers’ subjective constructions of their care provision experiences are reflective of concerns echoed in the literature outside of Indonesia and bring forth the overarching and ongoing healthcare issues around the world.

The highlighting of resource challenges and systemic deficiencies to which healthcare workers are subjected calls attention to the many facets of healthcare management in low-resource, crisis settings. In contrast to China amid COVID-19, for example, which deployed experienced experts to the COVID-19 battlefield [16], Indonesia’s COVID-19-designated hospital recruits its personnel through voluntary participation and military as well as police assignments. With the likelihood that many of its personnel are not always experts but are assigned merely because they are needed, there is a deep resignation due to (to name a few) the realization of having no control over the system, which has contributed to its high turnovers and many other manpower issues.

While the issue of human resources impacts healthcare workers in multifaceted ways, it is especially notable in terms of burnout and heavy workload, as exemplified by the instance of psychologists and nurses in the COVID-19 swabbing department. Service-induced burnout, as noted by Steel et al. [17], influences healthcare workers’ job satisfaction and turnover probability relative to both demanding workload and a lack of resources. However, the present study highlights the particular complexities of intersecting heterogeneous factors (e.g., high turnovers, demanding workload and burnout, and manifestations of altruism-motivated fears) and the hospital’s community quarantine policy during service contracts.

Despite adherence to the policy represents embodiments of psychological resilience [18] and a strong professional identity [5], social disconnections can affect the development of negative emotions, as in the case of healthcare workers’ resentment and disappointment over their conflicting moral obligations in the face of social discrimination and stigmatization. Yet, there are also times in which remoteness from the outside world can derive positive implications, such as healthcare workers fostering a sense of kinship and interconnectedness with patients despite not being blood-related. As seen in the study, recurrent and relational social relationship between healthcare workers and patients produces positive effects of sociality, enabling the amelioration of isolation-related negatives by relating to patients and their shared experiences.

There is, however, an obvious need to consider broader factors when discussing the interactions between healthcare workers and patients, and especially the impinging repercussions of healthcare rationing. In many ways, healthcare rationing is a “wicked problem” within which moral tensions reside at the intersections between efficacy and equity in healthcare [19], and its pervasiveness is at the center of COVID-19 and impacts the pandemic management from both top-down and bottom-up approaches. In the local experience of Indonesia, there are many productions of politicized tensions relative to the COVID-19 management, with the prioritization for economic interests over healthcare considerations affecting healthcare workers and their resource scarcity and challenges at (particularly) the COVID-19-designated hospital.

While the ramifications of having constrained resources and man-made capabilities have contributed to its dilemmatic healthcare rationing, it cannot be projected as the primary and sole cause when billions of Rupiahs have been allocated for the hospital. The inclusion of patients and their autonomy in terms of the ethical challenge posed by healthcare rationing also invites wider structural changes for engaging their wishes into meaningful patient care. Yet, even after *“a carefully considered advance directive”* [20], there was, in fact, blame externalization from patients’ emotion regulation reactions in what they perceive to be an unjust family member death *(“…A few days later, the family sent me a message stating, ‘Thank you for your advice. My family has died.’”)*. To the extent that healthcare rationing is an inevitable part of clinical practice and personal coping mechanisms can be helpful, there are also many pointing to the complex constellations of politics and patients’ autonomy that affect healthcare workers’ moral dilemma and complexity.

In one narrative, the consequence of healthcare rationing evokes the emergence of negative emotions when caring for their COVID-19-infected patients, such as circulating fears of being blamed by patients for malpractice, anxieties with the what-ifs, and doubts about their professional competence. Indeed, as evidenced by Fallman et al. [21], healthcare workers’ deficits in reciprocity and participatory, transparent decision-making imply that patients’ circumstances and outcomes are not always or solely dependent on themselves alone, but also on asymmetrical relationships [22], as well as insufficient professional autonomy and systemic rigidity [23]. Coping, consequently, becomes a prominent reparative measure for healthcare workers, with many of them citing religion and spiritual mending and healing as a primary strategy.

What is particularly emphasized with the many excerpts of religious coping is how it affords the meaning-making of finding a purpose in life through professional contributions to society. While, as noted by Wrzesniewski et al. [24], *“The modern sense of ‘calling’ may have lost its religious connection”*, they nonetheless credited it as an inspiring means for allowing individuals to enjoy their time, by describing, *“Work that people feel called to do is usually seen as socially valuable – an end in itself”*. In the case of healthcare workers, even at the expense where their concerns for others are at personal price (e.g., risking themselves for being infected), healthcare workers still perceive that their “other-oriented calling” [25] offers them with their perceived ethical ends of self-satisfaction and a sense of achievement.

In their particular expressionistic use of the Javanese proverb, *“Urip iku urup”* (Life is lit), healthcare workers are able to picture their moral worth based on their pronounced commitment to “calling” in caring for COVID-19 patients. In this, these fragments of calling-related professions point to derivations beyond just prosocial personality traits, altruistic attitudes, public service motivation, or other “generic” patterns of behaviors. That is, their humanitarian callings extend the perception of meaning-making in life purposes and project to navigating “profession of medicine” [26], within which to draw on Gustafson [27], *“A sense of calling… can affect one’s sense of worth and well-being as a professional person not only by assuring some dignity but by nourishing and confirming the deep moral motives that lead persons to professions”*. Indeed, notions of callings and service through the channeling of professions are not only about acts of which right things to do or which services benefit “the self” or “the others”, but rather indicative to framings of collective sense of morality and the impacts felt on these individuals, such as healthcare workers understanding their professional self-worth based on respective moral codes.

### Study strengths, limitations, and future directions

A strength of this study rests in its hermeneutic phenomenological methodology as it enabled the opportunity to achieve a more in-depth understanding than a superficial, qualitative analysis might have provided [28]. The analysis should not be viewed as conclusive and an absolute truth of the meaning of COVID-19 healthcare which would be out of step with the applied approach and also an illusion. The study, however, offers distinctiveness in providing insights into the perspectives and experiences of frontline healthcare workers in a COVID-19 central emergency hub, especially in contexts of resource constraint and political-institutional impediments in the Global South. While the study findings are reflective of the participants’ experiences, they cannot be extrapolated to all frontline healthcare workers in the sector; hence, the recruitment can be expanded to encompass a wider sampling base and potentially, more a more varied and nuanced spectrum of perspectives. However, it is believed that this study provides probable interpretations [14] that require readers to relate dialogically to the text to continue to improve understanding and expand the interpretive process, as well as to consider the relevance and transferability of the findings to other settings.

Since the hospital is largely governed by the Indonesian government and is operated by the military, there is a marked tendency to sidestep imperative but politically charged issues for fear of offending the government, including issues with healthcare workers not receiving financial compensation and facing consequences for speaking up (e.g., termination of employment). Such rendering gives avenues for future studies to include the perspectives of those from the governments in order to decipher the Indonesian politics of healthcare that has affected the grassroots levels and its broader population as to effect change and entice reform. Yet another great strength in this context is that the participants’ voices have been given a place in the article, so that they can be heard, even though the subject may be sensitive.

At the same time, the analysis and interpretation of the lived experiences and perspectives of frontline healthcare workers serving in Indonesia’s COVID-19-designated hospital has been carried out by an international collaboration between the Global South and North, with thus different preconceptions of and perspectives on healthcare infrastructure and practice. The collaboration not only brought together trustworthy perspectives, but also allowed for the exchange of critical thoughts and diverse perspectives, enabling the purveying of this article and enhancing its quality. Hence, it is hoped that this scholarly endeavour, which brings together a wealth of knowledge and interdisciplinary expertise, can be of service within and beyond the society.

### Healthcare implications

The present study encapsulates the lived experiences of healthcare workers functioning in a resource-limited and challenging setting, which has far-reaching implications for future public health crisis management as well as policy refinement. Although Indonesia is in the midst of a series of transitions, ranging from demographic and epidemiological, to social, economic, and political [29], the reverberation of infrastructure and facility-related challenges points to Indonesia’s historic fragmentation of underinvestment in its public health system, with a variety of contributing factors such as an absence of political commitment and a predilection for reactive interventions. In this, important notes should be on increasing the focus on the public health system as equal to economic considerations and political interests, for example, by improving human resource management and its resourcing system, as well as administering appropriate mental health solutions for service-related burnout and (especially) repercussions of healthcare rationing.

Undoubtedly, prioritizing the well-being of healthcare workers offers a distinct opportunity to advance health policy by acknowledging their indispensable role in the healthcare system, enhancing their working conditions, and investing in public health systems. Such an approach can ameliorate workforce shortages and augment patient outcomes by fostering a contented and stable healthcare workforce. Moreover, placing emphasis on healthcare worker welfare can culminate in long-term cost savings by curbing burnout and turnover rates, eventually translating into greater continuity of care and improved patient outcomes. Thus, acknowledging and prioritizing healthcare worker welfare in health policy can propel sustainable and effective healthcare systems, while ensuring the safeguarding of frontline healthcare workers, who are instrumental in delivering healthcare services.

## Data Availability

The datasets generated during and/or analyzed during the current study are available from the corresponding author on reasonable request.
